# Take a Look Towards the Stress Response of Working Dogs: Cortisol and Lactate Trend Mismatches During Training

**DOI:** 10.3390/ani15213175

**Published:** 2025-10-31

**Authors:** Raffaella Cocco, Sara Sechi, Giulia Sisia, Maria Luisa Pinna Parpaglia, Maria Rizzo, Federica Arrigo, Claudia Giannetto, Giuseppe Piccione, Francesca Arfuso

**Affiliations:** 1 Department of Veterinary Sciences, Teaching Veterinary Hospital, University of Sassari, Via Vienna 2, 07100 Sassari, Italy; rafco@uniss.it (R.C.); sarasechilavoro@tiscali.it (S.S.); pinnapar@uniss.it (M.L.P.P.); 2 Department of Veterinary Sciences, University of Messina, Polo University Annunziata, 98168 Messina, Italy; giuliasisia98@icloud.com (G.S.); federica.arrigo@studenti.unime.it (F.A.); claudia.giannetto1@unime.it (C.G.); gpiccione@unime.it (G.P.); farfuso@unime.it (F.A.)

**Keywords:** cortisol, blood lactate, working dog, stress response, training, welfare

## Abstract

The enrollment of working dogs in various fields is widespread throughout the world. Training adult working dogs could be stressful to dogs, which is why it is important to assess their response to these conditions. The aim of this study was to evaluate whether basic training in adult working dogs can be stressful. Cortisol and lactate concentrations were measured at three time points: T0, arrival at the training field; T1, immediately after training; and T2, 60 min after training. Stress-related behaviours were also recorded. None of the dogs showed behavioural signs of stress, such as yawning, barking, or stereotyped movements, although some displayed behaviours such as sniffing, licking their lips, keeping their ears back, or lifting their legs, which may also indicate stress. Cortisol concentrations were highest at T0, decreased at T1, and rose again at T2, while lactate concentrations increased at T1 and returned to baseline at T2. These findings suggest that the training itself was not a significant stressor, whereas the environment prior to training may have contributed to anticipatory stress.

## Simple Summary

The aim of this study was to evaluate serum lactate and cortisol levels in dogs during training. In particular, during the enrollment of working dogs, they may find themselves in stressful conditions and it is important to understand how the animals can adapt. Stress-related behaviours at T0 (arrival at the training field), T1 (immediately after training), and T2 (60 min after training) were analyzed in working dogs.

## 1. Introduction

Working dogs play a crucial role in numerous human activities, including security, search and rescue, and assistance [1]. Their performance depends not only on physical condition but also on psychological well-being. Therefore, understanding how training influences both stress and physical exertion is essential. The training process for these animals involves a combination of specialized approaches and procedures designed to harness their innate abilities and intelligence [2]. Dog training consists of teaching specific behaviours and commands to improve obedience, overall behaviour, and welfare during work. Properly conducted training minimizes the risks associated with potential hazards or challenging scenarios that working dogs may encounter [3]. It also strengthens the bond between dog and handler, facilitating effective communication and collaboration. A critical aspect of dog training is positive reinforcement, which can include treats, praise, or play, helping dogs understand expectations. Consistency is equally important; dogs learn best when cues and commands are clear and consistent [4]. Socialization is another essential component. By exposing dogs to various environments, people, and other animals, they learn to behave appropriately across diverse situations and in the presence of other dogs or different species. For working dogs, basic obedience commands, such as sit, stay, come, heel, and down, are of paramount importance [5]. These commands have been integral to working dog training for centuries. Historically, hunting dogs were trained to follow instructions, retrieve game, and assist in hunts. Over time, the applications expanded to include guarding, herding, and protection tasks [4]. Each command serves a specific function: (1) “sit” helps the handler control the dog’s movements and prevents approach to dangerous situations; (2) “stay” allows dogs to remain in place until further instructions, helping them maintain focus and avoid distractions; (3) “come” ensures reliable recall, even under challenging conditions; (4) “heel” teaches dogs to walk calmly beside the handler, maintaining focus during tasks; (5) “down” positions the dog low to the ground, which is useful in tasks requiring them to remain unobtrusive, such as search and rescue operations. Assessing a dog’s behaviour is crucial for assigning suitable working roles and monitoring welfare [5,6]. Individual behavioural differences contribute significantly to a dog’s suitability for specific tasks, often more so than physical traits. Extensive research has focused on identifying which behavioural traits facilitate success and which may hinder it [4,5,6,7]. A great deal of research has been focused on determining which specific elements of canine behaviour are positive and which are detrimental when it comes to the ability of working dogs to achieve their roles [2,4,5,6,7,8,9].

Achieving positive welfare involves not only protecting animals from negative experiences but also providing environments that enable positive experiences. Positive welfare encompasses happiness, quality of life, positive emotions, and affective engagement [10,11]. The ultimate aim is to safeguard animal welfare by minimize stressful or distressing experiences. Studying the behaviour of working dogs is a key component of preventive care, which seeks to maintain and enhance health while avoiding preventable problems.

Effective preventive care programmes for working dogs should include standard evaluations of health status of dogs alongside considerations of geographic location, breed, living and working conditions, and physical and mental demands [12]. Additionally, muscle fatigue and stress response during training must be considered, although it must be pointed out that not all muscle fatigue is bad, muscle strength is increased by exercising to fatigue. An animal’s ability to respond appropriately to stimuli that threaten homeostasis is vital for survival. Stress, whether physiological or psychological, triggers complex bodily responses aimed at restoring equilibrium and preventing pathology [13,14,15]. While training ensures operational effectiveness, it can also be a source of stress, particularly in challenging environments [5]. This stress response involves the hypothalamic–pituitary–adrenal (HPA) axis, leading to cortisol production. Measuring peripheral cortisol has been widely used as a welfare indicator to assess stress coping responses in therapy dogs [16,17,18]. During training, monitoring cortisol levels is particularly relevant, as concentration and moderate muscular work can influence its concentration [5,19,20,21,22,23,24].

Stress in dogs can be evaluated through both physiological and behavioural measures [19,20,21,22,23,24]. Cortisol is a well-known biomarker of stress; however, it has been suggested that the use of cortisol as a stress marker must be confirmed by behavioural observations, but, at the same time, it should be considered that symptoms of behavioural stress may not always positively correlate with cortisol production [24]. This implies the need to characterize the level of stress and to understand whether the stress response leads to a situation of discomfort for the animal. As a matter of facts, though being widely used in a negative sense, the term stress can also have a positive meaning. Indeed, two types of stress have been recognized, namely “eustress” or “good stress”, and “distress” or “bad stress”. While the distress leads to deleterious effect, the eustress is described as a non-threatening stress whose physiological indicators are excitement and increased level of arousal; this type of stress induces adaptive responses such as increased alertness, attention, and preparation for activity. Depending on the dog’s temper and/or the circumstance, the same type of stressor may induce a feeling of eustress, a mild and moderate distress, or a severe distress [24]. While cortisol is a well-established biomarker of stress, lactate levels indicate muscle fatigue following physical activity playing an important role in the limitation of exercise performance by declining muscle force or power output. However, this statement is currently too simplistic. As a matter of fact, during the past several decades, scientific thought has evolved and new understandings of the role of lactate in energy metabolism have altered this traditional theory [25]. It has been suggested that lactate has not confined to anaerobic conditions and that it has an important energy substrate that is readily utilized by multiple tissues throughout the body and it plays a crucial role in cell signalling during exercise [25].

Behavioural indicators, such as yawning, lip licking, and ear position, can also reflect stress in response to environmental challenges. Previous studies have explored stress in contexts such as veterinary procedures, kenneling, and environmental changes, but few have focused on routine training activities [19,20,21,22,23,24].

Based on this context, the present study aimed to investigate whether training affects blood lactate levels and serum cortisol concentrations in working dogs.

## 2. Materials and Methods

### 2.1. Animals

The study was conducted in accordance with Directive 2010/63/EU on the protection of animals used for scientific purposes, and the recommendations of the ARRIVE guidelines were taken into consideration. Ethical approval was obtained from the Body Responsible for Animal Welfare and Experimentation of the University of Sassari (OPBSA; Prot. no. 51237, 15 May 2025).

A total of 16 intact adult dogs (not neutered and/or spayed; no females in estrous were present during the study) were enrolled in the study (Table 1). All dogs had been trained on the same training field, undergoing two 15 min sessions per week in preparation for the IPO-1 (Internationale Prüfungs-Ordnung, Level 1) Working Trial. 

Dogs were included in the study if they satisfied the following criteria: owners provided informed consent for the scientific use of their animal’s data; clinically healthy during physical exam; free from external and internal parasites; and in good nutritional condition. All dogs lived in houses with free access to a private garden and were fed a high-quality commercial diet once daily according to body size and age. Prior to training sessions, dogs were fasted for 12 h. All dogs were trained by the same instructor and were handled by their owners during experimental procedures. All enrolled dogs had the same travelling experience; they were well accustomed to travelling and their last travel experience had occurred about 1 week before. A habituation period of 30 min was used after transport before the start of the training sessions. The animals arrived at the field with their owners and waited for their turn in confinement cages, without visual contact with other dogs but able to hear their vocalizations. Before entering the field, the owners took the dogs for a short walk to allow them to relieve themselves. A single experienced male behaviourist observed and videotaped each dog during the training sessions at the field (15 min). Each dog was observed for 30 min (from the time they exited the car until they returned to the car). Behaviour was recorded in real time using observation sheets filled out by three operators, assisted by a student in charge of video recording. The recordings were subsequently reviewed by a panel of four veterinarians to ensure the objectivity and reliability of the field observations. For each session, a structured form was completed to record behaviours related to stress and/or pacification. Behavioural data were collected using a continuous focal sampling method with all occurrences recorded. This allowed both the frequency and duration of specific behaviours to be quantified systematically across the training period.

Specifically, the following categories were considered. Stress-related behaviours, defined as indicators of physiological or psychological discomfort [26,27], included scratching, shivering, shaking, tail tucked between the legs, ears turned back, and vocalizations such as barking or whining. Pacification behaviours, also referred to as calming or appeasement signals [28,29], were defined as behaviours aimed at reducing tension in social or environmental contexts, and included lip licking and, in some contexts, sniffing when not related to environmental exploration. Lip licking was considered a pacifying behaviour when occasional, but excessive or repetitive lip licking was interpreted as a stress-related behaviour, consistent with previous literature on canine communication [26,27,28,29,30].

All dogs underwent a standardized aerobic training protocol (lasting 15 min), consisting of three sequential training exercises:

Exercise 1: From a sitting position, dogs walked 50 steps forward and backward, followed by 10 normal steps, 10 running steps, 10 slow steps, 10 normal steps, 10 steps with a sit command, and 10 steps with a lie-down command. Dogs then retrieved a 750 g wooden dumbbell placed 4 m away and returned it to the stationary owner.

Exercise 2: Dogs jumped over a 100 cm obstacle from a sitting start, ran to retrieve a 750 g wooden dumbbell positioned 3 m behind the obstacle, and returned it to the owner after a second jump over the obstacle.

Exercise 3: Dogs climbed a 180 cm fence from a sitting start, ran to retrieve a 750 g wooden dumbbell positioned 4 m behind the fence, and returned it to the owner after climbing the fence a second time.

All training sessions were conducted by the dogs’ regular handlers under the supervision of the same instructor, using positive reinforcement techniques (e.g., verbal praise and food rewards). Heart rate was monitored using Polar Vantage XL (Polar, Inc., Stanford, CN, USA) monitors to assess basal, average, and maximum heart rates during training.

### 2.2. Blood Sample Collection and Laboratory Analysis

**Figure 1:**
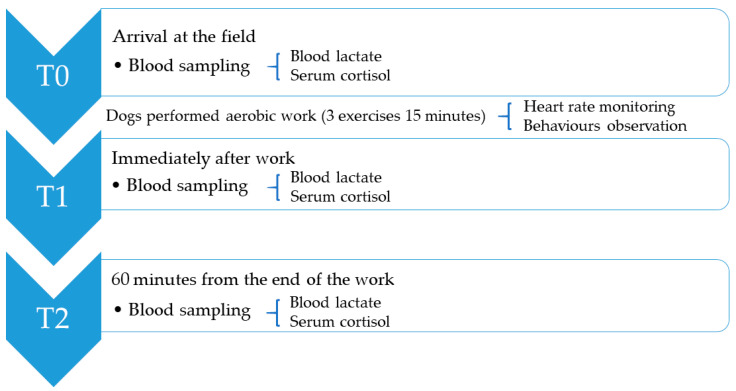
Schematic representation of the study protocol indicating the sampling and/or observation times.

Upon their arrival at the field (T0), immediately after training (within 1 min from the end of training, T1) and 60 min from the end of the training (T2) (Figure 1), blood samples were collected from all dogs from the cephalic vein of the forearm by applying a tourniquet into 2 types of tubes vacutainer tube containing EDTA (Terumo Co, Tokyo, Japan) and whey vacutainer tubes (Terumo Co, Tokyo, Japan). All dogs were habituated to blood collection and received positive reinforcement, such as treats and praise, during both training sessions and the actual blood draws to minimize stress. After collection, blood collected into EDTA tubes was immediately tested for blood lactate concentration by means of a small handheld meter (Accutrend Plus, Roche Diagnostics, Rotkreuz, Switzerland) [31,32,33,34], whereas blood samples collected into whey vacutainer tubes were cooled in an ice-water bath and transferred in the laboratory for the analysis. Samples were allowed to clot overnight at 4 °C before being centrifuged for 15 min at 1000× *g* in order to obtain serum. The obtained sera were tested for the concentration of cortisol by a commercially available ELISA kit (MyBioSource, San Diego, CA, USA, catalogue no. MBS703711; intra-assay coefficients of variation, <8%; inter-assay coefficients of variation, <10%; sensitivity, 1.56 µg/dL) by means of a ELISA microplate reader (BK-EL10B, BIOBASE, Shandong, Co., Ltd., Jinan, China). All calibrators and samples were run in duplicate and samples exhibited parallel displacement to the standard curve for both ELISA analyses.

### 2.3. Statistical Analysis

Data analysis was performed using GraphPad Prism v. 9.0. Normality was assessed using the Shapiro–Wilk test. Non-normally distributed data (*p* > 0.05) were analyzed using the Kruskal–Wallis test, followed by Dunn’s multiple comparison test for post hoc comparisons. The association between cortisol and lactate levels throughout the monitoring period was evaluated using Spearman correlation and linear regression; assumptions for regression (normality of residuals, homoscedasticity) were checked via Q–Q plots. Behavioural parameters recorded during training sessions were analyzed descriptively only, reporting frequencies and percentages of observed behaviours related to stress and pacification.

## 3. Results

The results of the present study are expressed as mean ± standard deviation (±SD). Average heart rates ranged from 141 to 179 bpm, with peaks of 180–240 bpm, indicating a normal cardiac response of investigated dogs to working conditions.

**Figure 2:**
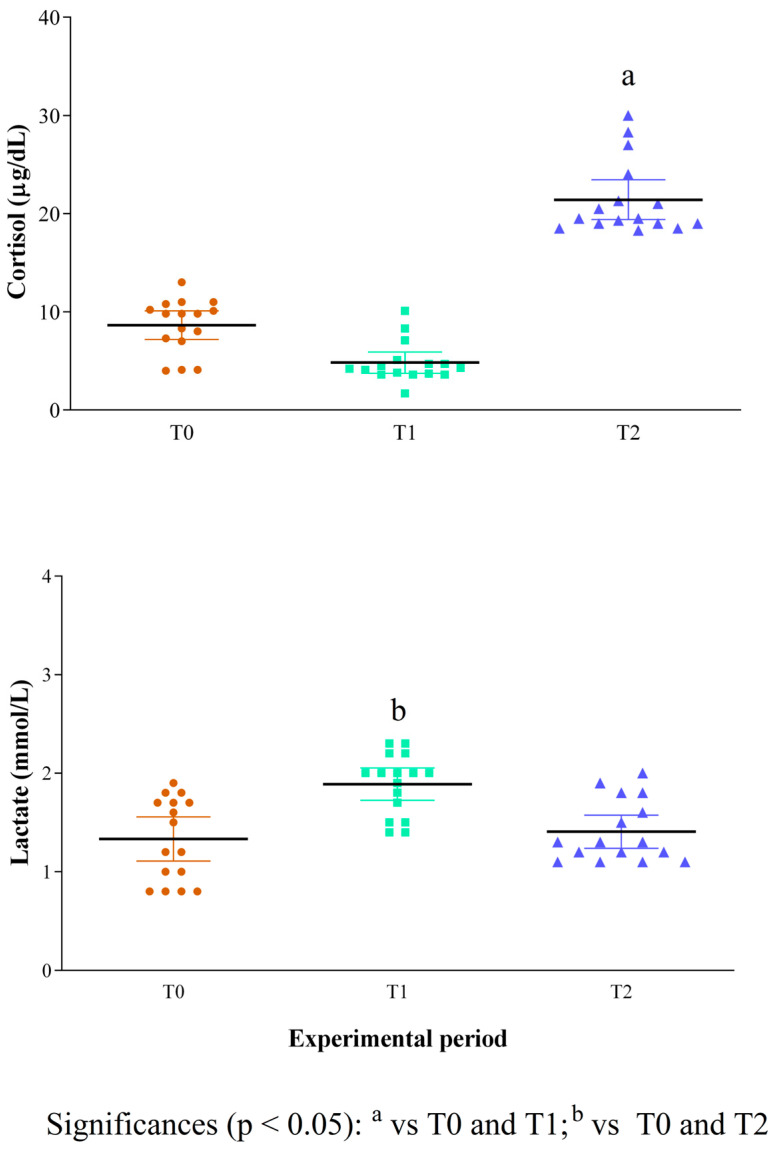
Mean values ± standard deviation (±SD) of serum concentration of cortisol and blood lactate measured in 16 working dogs during the experimental period (upon their arrival at the field, T0; within 1 min from the end of the work, T1; 60 min from the end of the work (T2). Normal reference ranges: cortisol, 0.96–6.81 µg/dL; lactate, 0.22–1.44 mmol/L) [32].

**Figure 3:**
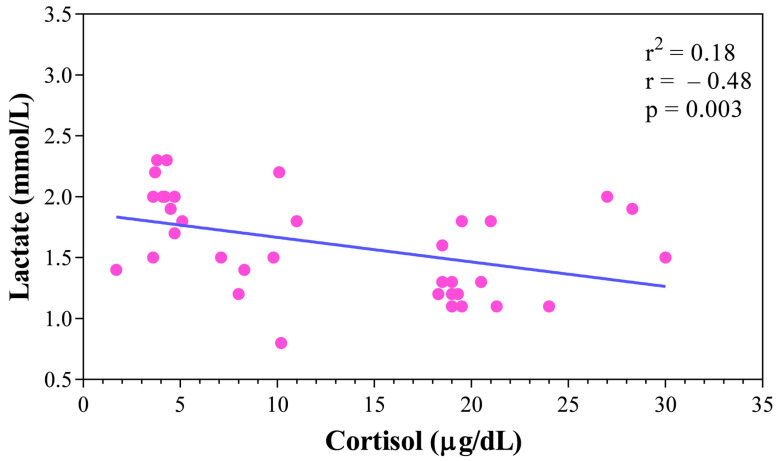
Relationship between cortisol and lactate concentrations measured in 16 working dogs throughout the experimental period.

Descriptive analysis of behaviours related to stress and pacification observed during the training sessions is reported in Table 2. Behavioural data were only collected at T1; therefore, no comparisons with T0 and T2 were possible. Stress-related behaviours, such as vocalizations, ears turned back, and tail tucked between the legs, were observed in a subset of dogs, while pacification behaviours, including lip licking, were the most frequently recorded. According to the statistical analysis results, the serum concentration of cortisol was higher at T2 than T0 and T1 (*p* < 0.05, Figure 2), whereas the concentration of blood lactate was higher at T1 than T0 and T2 (*p* < 0.05, Figure 2). A significant negative correlation between the concentrations of the serum cortisol and of the blood lactate was observed across the monitoring period, as confirmed by Spearman correlation and linear regression analysis (Figure 3), suggesting an inverse relationship between these two physiological stress markers in working dogs during the training sessions.

## 4. Discussion

The present study investigated physiological and behavioural stress responses in working dogs during standardized training sessions. Overall, the findings suggest that the pre-training environment and management, rather than the low-intensity exercise itself, were the primary sources of stress. Specifically, anticipatory stress was evident, with dogs displaying elevated cortisol levels even before training began (T0) compared to values obtained after exercise.

From a behavioural perspective, chronic stress can lead to abnormal or exaggerated behaviours, including stereotypies, which are detrimental as they can result in self-harm or ingestion of non-food substances [35]. In working dogs, stress can manifest as excessive barking, whining, or other behaviours indicative of psychophysical tension, especially when aversive training tools such as electric or spike collars are used [36]. Monitoring these behaviours, along with physiological markers, has become essential in safeguarding the welfare of working dogs [37,38].

Behavioural observations in the current study were categorized according to stress-related and pacification behaviours. Stress-related behaviours—scratching, shivering, shaking, tail tucked, ears turned back, vocalizations—reflect physiological or psychological discomfort [39,40]. Pacification behaviours—lip licking and sniffing not linked to exploration—reflect attempts to reduce tension in social or environmental contexts [28,29]. None of the dogs exhibited scratching, shivering, or shaking, but 14 of 16 licked their lips, 2 kept their tails tucked, 14 held ears turned back, and 10 vocalized. These behaviours indicate fear, stress, or mild aggression, consistent with findings that behavioural stress indicators often co-occur with elevated cortisol [17]. It should be noted that lip licking may serve different functions depending on frequency and context: occasional lip licking can be interpreted as a pacifying signal in response to adverse stimuli, including loud noises or restraint [41], whereas excessive or repetitive lip licking is more consistent with a stress response, particularly when associated with confinement or anticipation. Physiological responses further underscored the influence of the environmental conditions. The behaviour picture observed in enrolled dogs suggests a condition of mild–moderate stress, mainly related to the anticipatory and managerial component rather than to physical activity. These observations are in agreement with previous studies [26,27] that describe lip licking and low postures as typical signs of acute stress in dogs. Furthermore, the behaviour observations of the current study confirm what was highlighted in a previous investigation [5], according to which the training method profoundly influences the emotional and physiological response: the use of positive reinforcement, adopted in the present study, limits the manifestations of stress compared to aversive methods. Cortisol levels were above the normal range (0.96–6.81 µg/dL) in 6 out of 11 German Shepherds and all 5 Rottweilers at T0, prior to training [32]. This elevation likely reflected negative experiences associated with the training location, confinement in adjacent cages, and lack of social communication suggesting an anticipatory stress. Interestingly, cortisol dropped immediately after training (T1), suggesting that physical activity may serve as a temporary “relief valve,” providing an outlet for behavioural activation such as movement or play, which can reduce stress [42]. This finding highlights the role of exercise not merely as a physical activity but as a mechanism to alleviate anticipatory stress induced by pre-training management and the environment [43]. However, cortisol rose again at T2 (60 minutes’ post-exercise), likely due to prolonged confinement and limited recovery opportunities. During this phase, dogs were handled by their owners and confined while waiting for blood collection, with limited opportunities for social interaction or recovery. This context may explain the delayed increase in cortisol, reflecting environmental and management-related stress rather than exercise itself. Behavioural observations, including lip licking, tail tucking, ears turned back, and vocalizations, support this interpretation, indicating that while exercise temporarily mitigated stress, environmental and social stressors persisted post-training. It is also possible that at T1 some lip licking was influenced by exercise-induced salivation and fatigue, which may partially confound its interpretation as a stress marker. This pattern differs from previous studies [33,34,38] in which cortisol increased immediately after exercise and then declined within 30 min from the end of exercise, highlighting the significant contribution of emotional stress to HPA axis activation [39]. Peripheral markers of physical stress, such as blood lactate, showed a predictable trend: higher values immediately post-exercise (T1) and a return to baseline at T2. Lactate measurement was considered an important tool because it is known that its concentration in venous blood increases following moderate muscle work [31]. The shift towards anaerobic metabolism in skeletal muscle leads to an increase in lactate concentration [32,33,34]. The lactate trend observed in the current study, in agreement with the findings obtained in a previous study carried out on dogs performing a moderate aerobic exercise [44], seems to suggest that the physical load imposed by the exercises in the protocol did not result in significant muscle fatigue. This hypothesis is consistent with the observed mean (141–179 bpm) and peak (180–240 bpm) heart rate values, compatible with submaximal effort. The aerobic, low-intensity nature of the tasks (walking, trotting, obedience, retrieval, and jumping) likely limited muscular fatigue, explaining the modest lactate response. Notably, a significant negative correlation between cortisol and lactate suggests that central stress responses were primarily driven by psychological and environmental factors, whereas muscular stress followed expected metabolic patterns [44,45]. Furthermore, the negative correlation between cortisol and lactate could indicates that psychological stress does not necessarily follow the same trend as metabolic stress, as previously hypothesized in the context of integrated stress response mechanisms [40].

These findings highlight the complex interaction between emotional state, physiological stress responses, and observable behaviours. Cortisol mobilizes internal coping mechanisms, which in turn influence external behavioural responses, enabling dogs to meet the demands of challenging circumstances [12,40]. The anticipatory stress observed at T0 demonstrates that pre-exercise management and environmental context play critical roles in welfare outcomes. Exercise, while beneficial in reducing immediate stress, does not fully mitigate environmental and social stressors inherent in working dog training contexts. The collected data underline the importance of distinguishing between “anticipatory” stress and “exercise” stress when evaluating the welfare of working dogs. As underlined by other authors [21], success in selection and training programmes depends not only on the physical and cognitive abilities of the subjects, but also on their behavioural resilience and ability to manage the environmental and social context. Furthermore, a study conducted on German Shepherd dogs [46] confirmed the heritable component of behavioural traits such as sociability and stress management, strengthening the idea that selection should consider both physiological and temperamental markers.

Overall, the results emphasize the importance of combining behavioural observations with physiological measurements, such as cortisol and lactate, to obtain a comprehensive understanding of working dog welfare. Pre-training management procedures, including minimizing confinement stress and avoiding aversive training methods, are essential for reducing anticipatory stress and promoting long-term behavioural resilience.

## 5. Conclusions

The results obtained in the present study contribute to a better understanding of stress responses in working dogs during training sessions. Changes observed in serum cortisol and blood lactate concentrations reflected the hormonal and metabolic adaptations associated with physical exercise. In particular, the higher resting cortisol levels found in some dogs suggest a mild stress response prior to training, likely related to temporary confinement in cages. This interpretation is further supported by the rise in cortisol levels observed one hour after the end training, which exceeded typical physiological ranges. These findings emphasize the importance for handlers to recognize and respond to subtle signs of stress in their dogs, both during work and periods of inactivity, while considering that physical health and nutrition are closely linked to psychological welfare. However, this study presents several limitations including the sample size of investigated dogs’ group as well as the absence of a control group, the collection of behavioural data performed only at T1 limiting the ability to fully assess the dynamics of stress-related behaviours over time. Therefore, future studies including larger and more balanced samples, incorporating systematic behavioural observations at all phases of the protocol, and adding physiological indicators such as heart rate variability or immune function would be useful to better understand the relationship between physiological and behavioural stress responses in working dogs and to provide a more robust and integrated evaluation of working dog welfare.

## Figures and Tables

**Table 1:** Signalment data (i.e., breed, age, gender) for the 16 enrolled dogs.

n	Breed	Age	Gender	n
11	German Shepherd	Median 2 years	Males	7
		Range 1–6	Females	4
5	Rottweiler	Median 2 years	Males	2
		Range 2–6	Females	3

**Table 2:** Frequency of behaviours observed immediately after training (within 1 min from the end of training, T1). Data are presented as the number of occurrences (*n*) and the percentage of dogs (%) displaying each behaviour (*n* = 16) during the 15 min of training.

Observed Behaviour	*n* (%)	Behaviour Manifestation Time
**Body expression**		
Scratching/Sniffing/Shivering/Shaking	0 (0.0%)	-
**Mouth expression**		
The dog licks his lips	14 (87.5%)	Sit, lie down, approach the owner
**Tail language**		
The dog’s tail is between the legs	2 (12.5%)	Sit, lie down, approach the owner
**Ear appearance**		
The dog’ ears are turned back	14 (87.5%)	Sit, lie down, approach the owner
**Vocalization**		
The dog barked or whined	10 (62.5%)	Sit, lie down, approach the owner

## Data Availability

Data is contained within the article.
