# Correction: Cocco et al. Take a Look Towards the Stress Response of Working Dogs: Cortisol and Lactate Trend Mismatches During Training. *Animals* 2025, *15*, 3175

**DOI:** 10.3390/ani16050739

**Published:** 2026-02-27

**Authors:** Raffaella Cocco, Sara Sechi, Giulia Sisia, Maria Luisa Pinna Parpaglia, Maria Rizzo, Federica Arrigo, Claudia Giannetto, Giuseppe Piccione, Francesca Arfuso

**Affiliations:** 1Department of Veterinary Sciences, Teaching Veterinary Hospital, University of Sassari, Via Vienna 2, 07100 Sassari, Italy; rafco@uniss.it (R.C.); pinnapar@uniss.it (M.L.P.P.); 2Department of Veterinary Sciences, University of Messina, Polo University Annunziata, 98168 Messina, Italy

## References

In the original publication [1], there was a mistake in Ref. [28]. We corrected it in the list below. The authors state that the scientific conclusions are unaffected. This correction was approved by the Academic Editor. The original publication has also been updated.

The Reference 28: “Mariti, C.; Ricci, E.; Zilocchi, M.; Sighieri, C.; Gazzano, A. Human-directed social behaviour in dogs: The role of social experience. *Appl. Anim. Behav. Sci.* **2012**, *140*, 108–116” is not correct. It should be changed with “Mariti, C.; Gazzano, A.; Moore, J.L.; Baragli, P.; Chelli, L.; Sighieri, C. Perception of dogs’ stress by their owners. *J. Vet. Behav.*
**2012**, *7*, 213–219.”.
